# Role of purinoreceptors in the release of extracellular vesicles and consequences on immune response and cancer progression

**DOI:** 10.1016/j.bj.2024.100805

**Published:** 2024-11-05

**Authors:** Thomas Duret, Mohammed Elmallah, Jérôme Rollin, Philippe Gatault, Lin-Hua Jiang, Sébastien Roger

**Affiliations:** a Université de Tours, Inserm UMR1327 ISCHEMIA « Membrane Signalling and Inflammation in Reperfusion Injuries », Tours, France; b Service d’Hématologie-Hémostase, CHRU de Tours, Tours, France; c Service de Néphrologie, Hypertension, Dialyse et Transplantation Rénale, Tours, France; d Fédération Hospitalo-Universitaire Survival Optimization in Organ Transplantation (FHU SUPORT), Tours, France; e School of Basic Medical Sciences, Xinxiang Medical University, Henan, China; f School of Biomedical Sciences, University of Leeds, Leeds, United Kingdom

**Keywords:** Purinoreceptors, Extracellular vesicles, Exosomes, Cancer, Immune response

## Abstract

Cell-to-cell communication is a major process for accommodating cell functioning to changes in the environments and to preserve tissue and organism homeostasis. It is achieved by different mechanisms characterized by the origin of the message, the molecular nature of the messenger, its speed of action and its reach. Purinergic signalling is a powerful mechanism initiated by extracellular nucleotides, such as ATP, acting on plasma membrane purinoreceptors. Purinergic signalling is tightly controlled in time and space by the action of ectonucleotidases. Recent studies have highlighted the critical role of purinergic signalling in controlling the generation, release and fate of extracellular vesicles and, in this way, mediating long-distance responses. Most of these discoveries have been made in immune and cancer cells. This review is aimed at establishing the current knowledge on the way which purinoreceptors control extracellular vesicle-mediated communications and consequences for recipient cells.

## Highlights

• Extracellular nucleotides are key actors of cell signalling through P2 receptors.
• Dysregulated vesicle release is implicated in immune diseases and cancer.
• P2 receptors emerge as a mechanism controlling micro- and nano-vesicle release.

## 1 Purinergic signalling and cell communication

The concept of purinergic signalling was proposed by Geoffrey Burnstock, more than fifty years ago and was demonstrated to be responsible to what was initially called the “non-adrenergic, non-cholinergic nerve stimulation” [1,2]. While this signalling pathway initially faced strong scepticism, it is nowadays a fully established cell-to-cell communication pathway recognized for its strong significance, not only in the peripheral nervous system, in both physiological and pathological conditions. Indeed, nucleotides, most importantly adenosine 5′-triphosphate (ATP), but also adenosine 5′-diphosphate (ADP), uridine 5′-triphosphate (UTP), uridine 5′-diphosphate (UDP) and UDP-glucose, are released to the extracellular space in response to various stimuli such as mechanical pressure or distortion, electrical stimulation, osmotic swelling, hypoxia and ischemia, cytotoxic attacks and cell disruption [[3], [4], [5]]. These nucleotides could also be released following cell injury or through non-cytolytic pathways, such as exocytosis or even through plasma membrane channels or transporters [6]. Nucleotides, notably ATP, are ubiquitous extracellular signalling molecules, binding to and activating membrane-spanning receptors of the P2 family [5]. All mammalian cells express a panel of different P2 receptors having a critical role in physiology and basal tissue homeostasis by modulating essential cellular parameters such as cell proliferation and apoptosis, cell differentiation, migration or invasiveness. In contrast, dysregulation of this signalling pathway contributes to the pathogenesis of various diseases, including inflammatory, neurodevelopmental, neurodegenerative, behavioural and cancerous diseases [[7], [8], [9], [10], [11], [12]].

P2 receptors are categorized into two distinctive subfamilies in mammalians: ATP-gated ion channel P2X receptors, for which seven genes (*P2RX1-P2RX7*) encode seven corresponding P2X proteins (P2X1-P2X7) [13], and G-protein-coupled P2Y receptors, for which eight different genes (*P2RY1, P2RY2, P2RY4, P2RY6, P2RY11-P2RY14*) encode for P2Y proteins (P2Y_1_, P2Y_2_, P2Y_4_, P2Y_6_, P2Y_11_–P2Y_14_) [1]. P2X receptors are made of three P2X proteins subunits that can be of the same type (homotrimers) or composed of two different types of subunits (heterotrimers). P2X receptors all function as ATP-gated cationic non-selective channels, permeating K^+^, Na^+^ and Ca^2+^ and showing varied sensitivities to extracellular ATP (eATP) and different rates of desensitization following activation [14,15]. As a result, activation of the P2X receptors causes membrane depolarization and elevates the intracellular concentration of Ca^2+^ [16], which in turn activates additional transduction pathways such as mitogen-activated protein kinases (MAPK) and Ca^2+^-calmodulin-dependent enzymes.

Among all P2X receptors, the P2X7 receptor has attracted considerable attention regarding its structural and functional specificities, such as a molecular size higher than all other P2X receptors due a long intracellular carboxy-terminus end allowing specific protein-protein interactions, its unique homo-trimeric assembly and its strikingly low sensitivity to eATP. Indeed, while other P2X receptors are activated with micromolar concentrations of eATP, the activation of the P2X7 receptor (initially called cytolytic P2Z receptor [17]) requires hundreds or thousands of micromolar eATP, a condition that arising in few situations such as cytotoxic cell death [18]. The activity of the receptor is also characterized by its facilitation process and its ability to induce the formation of large pores across the plasma membrane that allow the passage of organic cations up to 900–1000 Da initially associated with cell death [17,[19], [20], [21]]. The P2X7 receptor is expressed in a variety of tissues and cells, but is most extensively studied for its crucial role in mediating immune and inflammatory responses and, when dysregulated, in participating in numerous inflammatory diseases [22,23]. The decrease in intracellular K^+^ concentration caused by the P2X7 receptor activation is a strong stimulus for activation of the NLRP3 inflammasome and the subsequent maturation and release of the potent pro-inflammatory cytokines, interleukin (IL)-1β and IL-18 [24]. Of interest to this review is the initial hypothesis that P2X7 receptor activation was responsible for release of IL-1β through microvesicle shedding and budding [[25], [26], [27], [28]].

P2Y receptors are G-protein coupled metabotropic receptors comprised of eight members in humans. Since several different nucleotides are possible agonists for P2Y receptors, they can be subclassified according to their preferred activating ligands as follows: the ATP-selective receptor (P2Y_11_), the ADP-preferring receptors (P2Y_1_, P2Y_12_ and P2Y_13_), the UTP-preferring receptor (P2Y_4_), the UTP- and ATP-preferring receptor (P2Y_2_) and the UDP-glucose or UDP-galactose-preferring receptor (P2Y_14_). Nevertheless, high concentrations of ATP can also activate P2Y_1_ and P2Y_13_ receptors, or inhibit P2Y_4_ and P2Y_12_ receptors.

Associated intracellular signalling pathways mostly depend on the type of G-protein these receptors are coupled to. P2Y_1_, P2Y_2_, P2Y_4_ and P2Y_6_ receptors mainly activate the G_q/11_ protein, thus inducing phospholipase C-β (PLC-β)-dependent generation of intracellular second messengers, inositol 1,4,5-triphosphate (IP_3_) and diacylglycerol, which activate the IP_3_ receptors and release of endoplasmic reticulum Ca^2+^ to increase intracellular Ca^2+^ and protein kinase C (PKC), respectively. The P2Y_11_ receptor is coupled to the G_q_-protein, triggering an increase in intracellular Ca^2+^ level through an IP_3_ receptor-mediated release of Ca^2+^ from the endoplasmic reticulum, or alternatively the G_s_-protein, activating adenylyl cyclase (AC) to generate cyclic adenosine monophosphate (cAMP). By contrast, P2Y_12_, P2Y_13_ and P2Y_14_ receptors are mainly coupled to the G_i_-protein, thus inhibiting AC and lowering the intracellular cAMP concentration [29]. Apart from these conventional pathways, recent studies have described the activation of additional intracellular signalling pathways activated by P2Y receptors, through the recruitment of Gβɣ subunits, such as phosphatidylinositol-4,5-biphosphate 3-kinase ɣ (PI3K-ɣ), phospholipase C-β2 and –β3, G protein-coupled receptor (GPCR) kinase 2 and 3, Rho GTPases and MAPK [30].

The purinergic signalling pathway is delicately and finely regulated in space and time. Indeed, nucleotides show relatively short half-life in the extracellular compartment. This is principally due to their degradation by different types of enzymes, called ectonucleotidases, which are either soluble or attached to the cell surface. These ectonucleotidases hydrolyse nucleotides tri-, di-, and mono-phosphates into their respective nucleosides, with some specific selectivity on substrates. There are four major classes of ectonucleotidases: ***1)*** the ectonucleoside triphosphate diphosphohydrolases (ENTPDases, also referred as Ecto-Apyrases, NTPases or E-ATPases) including CD39, CD39L1 and CD39L3. ENTPDases also hydrolyse dinucleoside polyphosphates, ADP ribose and NAD^+^, but with a low affinity; ***2)*** the ecto-5′-nucleotidase (5′-NT), also known as CD73; ***3)*** the ectonucleotide pyrophosphatases/phosphodiesterases (ENPPS); and ***4)*** the alkaline phosphatases (APs) [31]. The soluble ENTPDase 1, also known as CD39, preferentially hydrolyses ATP into ADP then in AMP. AMP is further hydrolysed to adenosine by the membrane-bound CD73. This is the major source for the production of extracellular adenosine from AMP. CD73 can also hydrolyse other nucleotides mono-phosphates, such as UMP, CMP, GMP and IMP but with a lower affinity than for AMP. While there is no identified receptor activated by extracellular AMP, adenosine is a powerful ligand acting on membrane receptors (originally called P1 receptors) comprising four types of G protein-coupled receptors: A1, A2A, A2B and A3. The activation of these receptors by adenosine, triggers AC stimulation (A2A, A2B) or inhibition (A1, A3), thus increasing or decreasing intracellular cAMP concentration, respectively. Eventually, adenosine is further degraded to inosine by adenosine deaminase (ADA) [32,33]. These findings highlight the high plasticity of nucleotide-stimulated intracellular signalling, depending on the expression of multiple receptors and ectonucleotidases by cells, concentrations and half-life of ligands, activating successively or concomitantly multiple pathways responsible of potentially opposite responses. Considering the rapid degradation of extracellular nucleotides and adenosine, their action is limited in space and time duration.

Recent studies provide evidence to suggest that purinoreceptors are not only involved in autocrine or paracrine cell communication, for a short period of time, but may also be involved in long distances cell-to-cell communications and for a prolonged duration through the release of extracellular vesicles (EVs). While these findings originally came from studies performed in cancer and immune cells, in [Table I], it is most likely that such mechanisms are generalizable to other cell types, in physiological or pathological conditions.

**Table 1:** Role of purinoreceptors and ectonucleotidases in regulating extracellular vesicles and physiological/pathological consequences.

Receptors or enzyme involved	EVs type and source	Purinergic agonist/antagonist	Effect on EVs release and cargo	Physiological and pathological impacts	
P2Y_12_	Platelets-derived EVs	ADP agonist	↑EVs release	Pro-coagulation	[59]
		Ticagrelor antagonist	↓EVs release		
	Platelet-derived EVs	PSB-0739 antagonist	↓ EVs release		[60]
P2Y_1_	Platelet-derived EVs	MRS2179 antagonist	↓ EVs release		[59]
P2X7-dependent NLRP3 inflammasome activation	Macrophage-derived exosome	ATP agonist	Loading IL-1β in released exosome	Inflammation	[79,80]
	Macrophage exosome	ATP agonist	↑MHC-II containing exosome release	Inflammation	[81]
	Macrophage Exosome	ATP agonist	↑CD14 release of CD9^+^ EV	Inflammation	[82]
RILP cleavage dependent Caspase-1 activation-P2X7	Hela cells-derived exosomes		Loading specific AAUGC motif-containing miRNA in exosome	Inflammation	[85,86]
P2X7	Monocyte microvesicle	BzATP agonist	↑PS^+^ microvesicle release	Inflammation	[25]
	Macrophage-derived exosome	oATP antagonist	↓Exosome release	Inflammation	[98]
	Macrophage-derived exosome	ATP agonist A740003 antagonist	↑Exosomal TACE release	Inflammation	[99]
	Neuroblastoma cell line-derived EVs	BzATP agonist	ATP exocytosis		[106]
	Melanoma cells-derived exosomes/microvesicles	BzATP agonist A740003 antagonist	Increase of miR-495-3p, miR-376c-3p, and miR-6730-3p containing EVs	Pro-metastatic activity	[107]
	Small EVs of TAMR-MCF-7 cells	KN62 antagonist	↓EVs secretion	Cancer cell migration	[108]
	HuCC-T1-derived EVs	Hypermethylation of *P2RX7* gene	↓CD9^+^ and CD81^+^ EVs	Tumour progression	[110]
P2X4	HCV infected hepatocyte cells	ATP agonist	↑EVs release	HCV infection	[117]
CD73	Exosome derived from plasma of head and neck cancer patients		Release of EVs expressing surface-presenting CD73	Adenosine production	[124]
	U-87 MG glioblastoma cell line-derived exosomes		Release of EVs expressing surface-presenting CD73	T Cell aerobic glycolysis inhibition promoting GBM proliferation	[126]
CD39/CD73	HT376 bladder cancer cells		Loading CD73 and CD39 in released EVs	T Cell inhibition through adenosine production	[123]
	MCF7 breast cancer cell				
	Caco-2 colorectal cancer cell				
	DU145 and PC3 prostate cancer cell				
	Small EVs from MIA PaCa-2 pancreatic cancer cells and H1299 non-small cells		Release of EVs expressing surface-presenting CD73	Mast cell activation	[127]
	Exosome derived from plasma of head and neck cancer patients		Loading CD73 and CD39 in released EVs	Expression correlated with cancer stage	[125]
	UMSCC47 head and neck cancer cell line		Loading CD73 and CD39 in released EVs	Angiogenesis through adenosine production	[128]
	Microvesicles from bone marrow of multiple myeloma patients		Loading CD73 and CD39 in released EVs	Immunosuppression	[129]
A2A	U251 glioblastoma cell line, Mel526 metastatic melanoma cell line, MDA-MB-231 breast cancer cell line UMSCC47 head and neck cancer cell line Preglomerular vascular smooth muscle cells	CGS21680 agonist SCH442416 antagonist A2A KO	↓EVs release ↑ EV release ↑ EV release under basal end stress condition	Apoptosis of Jurkat T cell	[130]
A1	MDA-MB-231 breast cancer cell line Preglomerular vascular smooth muscle cells	CCPA agonist A1 KO	↓EVs release Constrain EV release under basal and stress metabolic condition		[130]
A3	Thyroid lobe culture Liver cell Lung cell	MRS1191 Antagonist MRS1191 Antagonist MRS1191 Antagonist	↓FADD containing EV release ↑FADD containing EV release ↑FADD containing EV release	Regulation of apoptosis	[130,131]
A2B	Thyroid lobe culture Liver cell	MRS1754 Antagonist MRS1754 Antagonist	↑FADD containing EV release ↓FADD containing EV release	Regulation of apoptosis	[130,131]
	Preglomerular vascular smooth muscle cells	A2B KO	Constrain EV release under metabolic stress condition		[130]

## 2 Different types of extracellular vesicles (EVs)

Extracellular vesicles comprise heterogeneous populations of vesicles in a nanometer and micrometer size including exosomes (30–200 nm in diameter), microvesicles (100–1000 nm) and apoptotic bodies (1–5 μm) [34]. Exosomes are small lipid bilayer nanovesicles generated in the endosomal compartment and released into the extracellular compartment upon fusion of multivesicular bodies (MVBs) with the plasma membrane. These vesicles are potentially released by all types of cells, however showing different molecular compositions related to their compartimental origin and environmental conditions. Exosomes carry a variety of biologically active components such as proteins, lipids, DNA, RNA, mRNA, miRNA and LncRNA. They are present in all body fluids. These cargos can be delivered to target cells either in the close vicinity or distant from their release, thus modifying their gene expression profiles as well as inducing multiple signalling pathways [35,36]. Therefore, exosomes have been reported to play a crucial role in intercellular communications including immune response [37], physiological signal transduction and antigen presentation [38], as well as pathological conditions such as the initiation and progression of chronic inflammation [39], cardiovascular and renal diseases [40] and cancer [41].

### 2.1 Biogenesis of small EVs

**Fig. 1:**
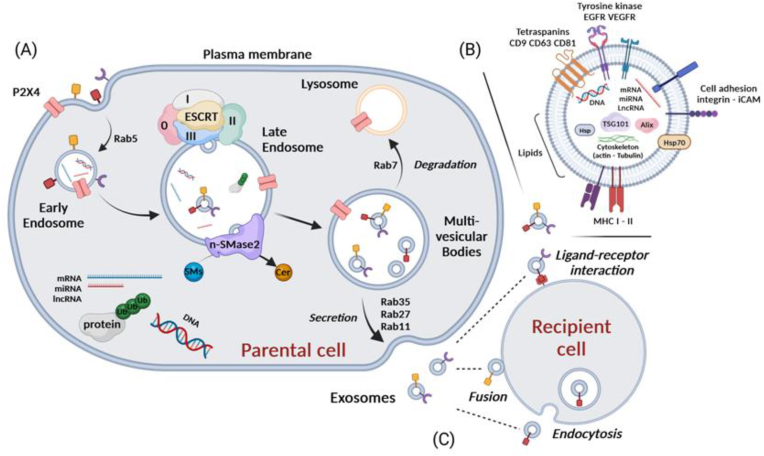
(A) Exosome Biogenesis occurs within the endosomal compartment. ESCRT-dependent pathway-mediated exosome biogenesis begins with the inward budding of the plasma membrane to form early endosomes (EE) which are then maturated into late endosomes (LE). ESCRT sub-complexes 0, I, II and III are recruited to the endosomal membrane and cooperatively orchestrate the sorting of protein cargo into the intraluminal vesicles (ILVs) and the formation of multivesicular bodies (MVBs). MVBs can either fuse to the lysosome for degradation or to the plasma membrane leading to the release of exosomes into the extracellular space. ESCRT-independent pathway occurs via specific lipid raft microdomains enriched with sphingolipids. The enzyme neutral sphingomyelinase 2 (nSMase2) converts sphingomyelin to ceramides which allow lateral phase separation and the abscission of ILVs from the endosomal membrane. (B) Structure and molecular composition of exosomes/Small EVs (C) Delivery of the released exosomes from parental cell to the recipient cell can be performed by three different mechanisms including ligand-receptor interaction, direct fusion of the lipid bilayer of exosome with the plasma membrane, or by endocytosis.

The process of exosome (belonging to the classification of small EVs) biogenesis occurs within the endosomal compartment [Fig. 1], beginning with the endocytic invagination of the plasma membrane to form early endosomes (EE), followed by the maturation of early endosomes into late endosomes (LE). Late endosomes tend to form a second inward budding and incorporate various cargo into intraluminal vesicles (ILVs) to generate MVBs [42]. The molecular mechanism by which the cytoplasmic cargo is incorporated into ILVs still remains a matter of debate. However, strong evidence suggests that a large protein machinery known as endosomal sorting complex required for transport (ESCRT) is involved in the generation of ILVs. ESCRT encompasses four different protein sub-complexes, called ESCRT-0, ESCRT-I, ESCRT-II and ESCRT-III. They are recruited to the late endosomal membrane and orchestrate cargo sorting, budding of ILVs and formation of MVBs [43]. Briefly, ESCRT-0 interacts with ESCRT-I and ESCRT-II to recognize ubiquitinated proteins, via their ubiquitin-binding units, and sequester them into endosomes. ESCRT-I and ESCRT-II facilitate the invagination of the late endosomal membrane. At the site of membrane budding, ESCRT-II combines with ESCRT-III, resulting in the abscission of ILV from the endosomal membrane and the formation of MVB. Following its formation, MVB can be either fused with lysosomes for degradation under the action of the Rab7 GTPase or fused to the plasma membrane to release exosomes into the extracellular space. Several Rab GTPases have been suggested to facilitate the fusion of MVB to the plasma membrane including Rab35 [44], Rab27 [45] and Rab11 [46]. The implication of the ESCRT-dependent pathway in exosome biogenesis has also been studied. Notably, the ESCRT protein components like ALIX (ESCRT-III) and TSG101 (ESCRT-0) are present in the released exosomes of different cell types [47]. In addition, ALIX has been shown to play a major role in loading, as well as in deciphering the composition of exosome proteins [48]. Others proteins such as transmembrane tetraspanins CD9, CD63, CD81 are particularly enriched in exosomes [49]. While CD9 is commonly present on the surface of exosomes, its use as a reliable marker for exosomes remains questionable since it is also present on the surface of a multitude of cell types and involved in cell adhesion and migration, platelet activation and aggregation, and immune cell activation [50]. CD63 is mainly associated with membranes of intracellular vesicles. It is considered as a marker for MVBs [51] as well as for extracellular vesicles released from the multivesicular body [49]. Alternatively, lipids were also reported to be involved in the formation and release of exosomes in an ESCRT-independent mechanism [52]. This mechanism is mainly linked to specific raft microdomains-enriched sphingolipids for the lateral segregation of ILVs from the endosomal membrane. It has been reported that the conversion of sphingomyelins to ceramides is mediated by the neutral sphingomyelinase 2 (nSMase2) on the late endosomal membrane. Ceramides are able to exert a lateral phase separation and coalescence, resulting in abscission of ILVs from the endosomal membrane [53]. Consequently, as demonstrated in a mouse oligodendroglial precursor cell line, inhibition of nSMase2 was shown to induce a significant reduction in the release of exosomes [54]. Finally, released exosomes can be received by target/recipient cells, influencing their physiological and phenotypic functions. The incorporation of exosomes in recipient cells can be achieved by different mechanisms showing some specificities. Some incorporations require specific receptor-ligand interactions, while others are realized by endocytic internalization or even by direct fusion of exosome lipid bilayer with plasma membranes of recipient cells membranes [55].

### 2.2 Biogenesis of large EVs

The biogenesis of microvesicles, or so-called large EVs (100–1000 nm), occurs through the plasma membrane budding followed by scission. Activation of the small GTP-binding protein ADP ribosylation factor 6 (ARF6) and subsequent rearrangement of the actin cytoskeleton are a key regulator of conventional microvesicle biogenesis [35]. Microvesicles are characterized by the presence of some molecular markers such as Annexin A1, A2 or α-Actinin 4. However, because of their high level of heterogeneity, some microvesicle populations can be generated via cellular mechanisms showing overlaps with those involved in exosome biogenesis [34]. Similarly, to the nSMase2 activity in exosome biogenesis, the acid-sphingomyelinase (A-SMase) pathway induces a ceramide-dependent microvesicle generation [56].

## 3 Role of P2Y1 and P2Y12 in EV release by platelets and consequences in cancer

Activated platelets are characterized by the secretion of a variety of substances, mostly involved in clot formation and wound healing, through microvesicles shedding or exosome release [57]. The ADP-sensitive P2Y_1_ and P2Y_12_ receptors are highly expressed in platelets. There is evidence that these two receptors play distinct roles in platelet activation and aggregation and in biogenesis and release of EVs. While the P2Y_1_ receptor is involved in platelet activation through the Gq/PLC/IP_3_-dependent elevation of cytosolic Ca^2+^ concentration, the P2Y_12_ receptor induces a decrease of intracellular cAMP concentration, through the inhibition of AC, thus increasing platelet sensitivity to platelet activation. Nevertheless, the action of both receptors is necessary for the biogenesis of EVs by platelets [58]. The pharmacological antagonism of P2Y_12_ receptor is the most effective treatment strategy to prevent platelet activation, clot formation and stent thrombosis after percutaneous coronary. Treatment with the P2Y_12_ receptor antagonist ticagrelor efficiently decreased release of EVs from ADP-induced pro-coagulant platelets, which was further enhanced by co-treatment with the P2Y_1_ receptor antagonist MRS2179 ^59^. Comparably, the release of EVs by platelets was inhibited after treatment with the P2Y_12_ receptor antagonists PSB-0739 ^60^.

Recently, some studies suggest close interconnection between platelet activation and cancer progression. Platelet EVs, also referred as microparticles (MPs), appear to take part in this process [61]. Indeed, in gastric cancer, the aggressivity of tumours and the development of metastases have been correlated with high levels of circulating platelet microparticles [62]. Furthermore, in non–small cell lung cancer (NSCLC) patients, platelet-derived MPs are significantly increased and have been shown to deliver multiple miRNAs, such as miRNA-223, promoting cancer cell invasion *in vitro* [63]. Platelet MPs have also been reported to stimulate MAPK in lung cancer cells and to increase cell proliferation, angiogenesis and invasion [64]. In breast cancer, platelets MPs can promote adhesion of mammary cancer cells to human umbilical vascular endothelial cells (HUVEC), through the transfer of CD41, and also induce chemotaxis, invasion and upregulate the production of matrix metalloproteases (MMP) such as MMP-2 and MMP-9 [65]. The role of the ADP-sensitive P2Y_1_ and P2Y_12_ receptors expressed on platelets was also assessed in the tumour growth of primary ovarian cancers [66]. It was elegantly demonstrated that pharmacological inhibition using Ticagrelor or general knockout of the P2Y_12_ receptor in mice (*P2ry12*^−/−^) importantly reduced the growth of syngeneic ovarian cancer tumours. Furthermore, reconstitution of haematopoiesis in irradiated *P2ry12*^−/−^ mice by wild-type hematopoietic progenitor cells restored tumour growth. Knockdown of CD39 ecto-apyrase on ovarian cancer cells increased tumour growth in tumour-bearing mice. Finally, in the absence of platelets, treatment with ADP, Ticagrelor or recombinant apyrase, or knockdown of CD39 did not affect ovarian cancer cell proliferation. In contrast, in the presence of platelets, treatment with Ticagrelor and recombinant apyrase reduced cancer cell proliferation. On the contrary, knockdown of CD39 increased cancer cell proliferation. These results provide convincing evidence for the critical role for ADP and platelet P2Y_12_ receptor for promoting primary ovarian cancer tumours in mice through platelets-cancer cells communication [66]. Nevertheless, it was not assessed whether this was mediated by EVs release. As demonstrated in another study, ADP-sensitive P2Y_1_ and P2Y_12_ receptors play a role in the release of angiogenic factor vascular endothelial growth factor (VEGF) from activated platelets [67]. In this study, antagonism of both P2Y_1_ receptor using by MRS2179 and P2Y_12_ receptor by Cangrelor inhibited platelet release of VEGF. Considering the high concentration of extracellular ADP in tumours, this has important consequences on tumour progression. Collectively, these results suggest that antagonism at P2Y_1_ and P2Y_12_ receptors, with clinically used drugs, might represent an interesting therapeutic strategy to prevent platelet-mediated tumour progression.

## 4 Role of the purinergic signalling and NLRP3 inflammasome activation in EV secretion

Inflammasome is a large cytosolic multiprotein complex that multimerizes and activates in response to microbial infection and stress-associated stimuli in order to trigger the inflammatory response [68]. Such a complex consists of a sensor protein from the family of pattern-recognition receptors (PRRs) for pathogen- or damage-associated molecular pattern (PAMPs or DAMPs, respectively), the adaptor protein apoptosis-associated speck-like protein containing a CARD (ASC), and procaspase-1. PRRs can be divided, based on their structure, ligands and the cellular compartments that they survey, into four families. The first two families consist of the plasma membrane-bound receptors for diverse microbial ligands, exemplified by the toll-like receptors (TLRs) and C-type lectin receptors. The third family are cytosolic, with the absent in melanoma 2 (AIM2) being an example. The fourth family of PRRs is also cytosolic and representative members include the nucleotide-binding and oligomerization domain (NOD)-like receptors, also known as nucleotide-binding leucine-rich repeat (LRR) receptors (NLRs) [69,70]. Cytosolic PRRs can alternatively be categorized into NLRs and non-NLRs; the NOD-, LRR- and pyrin domain-containing protein 1 (NLRP1), NLRP3, and NLR family caspase activation and recruitment domain (CARD)-containing 4 (NLRC4) are well-known NLRs, and AIM2 is a non-NLR.

Several factors have been identified for the canonical activation of the NLRP3 inflammasome, including *1)* stimulation of the transcription factor NF-kB upon binding of ligand/priming stimuli to the toll-like receptor (TLR) and NLR, *2)* increased level of eATP and activation of the P2X7 receptor mediating K^+^ efflux, Ca^2+^ and Na ^+^ influx [71], *3)* mitochondrial dysfunction and generation of reactive oxygen species (ROS), and *4)* lysosomal damage. Finally, NLRP3 allows the cleavage and activation of pro-caspase-1 into active caspase-1, responsible for the proteolytic maturation of both IL-1β and IL-18 pro-inflammatory cytokines [72]. Unlike most cytokines, IL-1β lacks a secretory signal sequence, raising the question on the secretory way(s) by which IL-1β is secreted by the producing cells. Several different pathways have been proposed for IL-1β secretion, most notably from monocytes and macrophages [73], which are the predominant cell types for its production, but also in neutrophils [74]. In the past 30 years, many secretory routes have been proposed, including exocytosis of secretory lysosomes or secretory autophagosomes, release through microvesicle shedding from the plasma membrane, exosomes secretion, or secretion through plasma membrane permeabilization occurring during pyroptosis [75,76]. Apart from the lastly mentioned route, the mostly accepted mechanism appears to be a vesicular pathway, even though the cellular compartment of origin and the size of EVs can be debated.

Ferrari and collaborators were the first to demonstrate that the P2X7 receptor was responsible for eATP-driven maturation and secretion of IL-1β [77]. Activation of the P2X7 receptor induces K^+^ efflux, leading to decrease of the intracellular K^+^ concentration which promotes inflammasome activation. In addition, activation of the P2X7 receptor mediates IL-1β secretion by inducing a sustained rise in the cytosolic Ca^2+^ concentration [78] and, nevertheless, there is evidence to indicate that activation of the P2X7 receptor by eATP promotes NLRP3 activation [79] and IL-1β secretion [80] from macrophages independently of Ca^2+^ mobilization. Dubyak and his team showed that the P2X7 receptor in macrophages and dendritic cells (DCs) was responsible for the release of two types of major histocompatibility complex class II (MHC–II)–containing vesicles from distinct biogenesis: one pool was comprised of microvesicles in a diameter of 100–600 nm and derived from direct budding of the plasma membrane, while the second pool was composed of exosomes in 50–80 nm and released from the fusion of MVBs with the plasma membrane. In addition, activation of the ASC/NLRP3 inflammasome by the P2X7 receptor was required for the release of MHC–II–containing exosomes, while other effectors such caspase-1 and possibly other proteases might be necessary [81]. Pelegrin and his group demonstrated that activation of the NLRP3 inflammasome, via the P2X7 receptor, induced the release of small size vesicles by macrophage during sepsis. These vesicles were expressing the CD9 marker and were containing CD14, a co-receptor of TLRs for the detection of pathogens [82]. The average size of these EVs was 167 nm, falling in the classical range of exosomes, but the exact nature of such EVs was yet not clearly defined.

In microglial cells, the P2X7 receptor is primarily known for its role in immune response, particularly activation of the NLRP3 inflammasome, and also participates tin the clearance of both extracellular and intracellular debris through modifications of lysosomal function, phagocytosis and autophagy. It has been reported that the stimulation of the P2X7 receptor with eATP impaired lysosomal functions through an intralysosomal pH increase, leading to a decrease of the autophagic flux that was accompanied with the extracellular release of autophagolysosomes [83]. These effects were mainly attributed to lysosomal leakage that also leads to a rise in cytoplasmic cathepsin B to induce activation of the NLRP3 inflammasome and subsequent maturation and release of IL-1β [84].

**Fig. 2:**
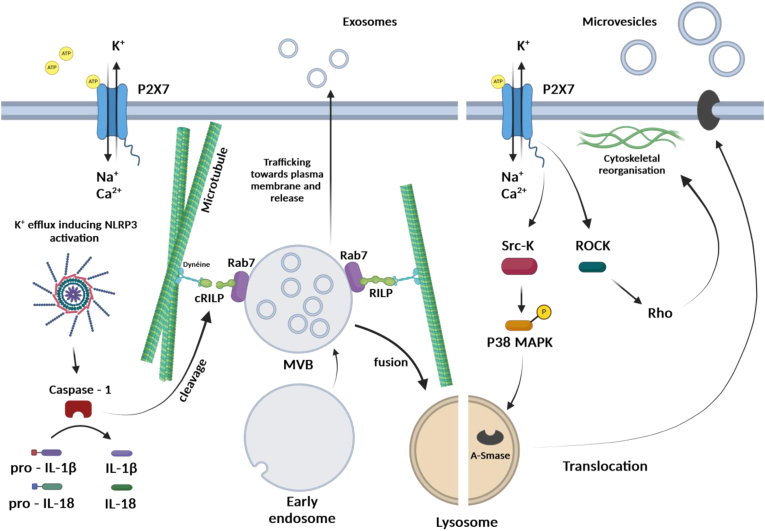
Small and large EV release from ATP-induced P2X7 stimulation. P2X7 activation and consequent K+ efflux promote NLRP3 inflammasome activation and caspase-1 maturation. Caspase-1 maturates Pro-Interleukin (IL)-1β and pro-IL-18 into IL-1β and Il-18, respectively, and cleaves RILP which participates to exosome release. P2X7 also recruits Src-Kinase, leading to P38 MAPK phosphorylation allowing the translocation of lysosomal A-SMase to the plasma. A-SMase produces ceramide from sphingomyelin which facilitates membrane blebbing and microvesicle release. In parallel, ROCK activation by P2X7 allows the reorganization of F-actin cytoskeleton which is indispensable for microvesicle release.

Cells are known to respond to inflammatory states often by an increase in exosome secretion. Nevertheless, the way by which inflammatory response alters cargo specificity and secretion of exosomes is unclear. Wozniack and collaborators have proposed that activation of the NLRP3 inflammasome and caspase-1 in human monocytes, upon stimulation with lipopolysaccharide (LPS) and eATP, induces exosome release through caspase-1-dependent cleavage of the Rab-interacting lysosomal protein (RILP) [Fig. 2]. RILP is part of a multiprotein complex that links the trafficking GTPase Rab7 to the dynein motor system, allowing fusion of MVBs with the lysosome. It has been shown that cleavage of RILP resulted in a truncated form, thereby inhibiting the fusion of MVBs with lysosomes and instead promoting their fusion to the plasma membrane. This was consequently promoting the release of exosomes into the extracellular milieu. Furthermore, RILP cleavage regulated the packaging of specific AAUGC motif-containing miRNA in the intraluminal vesicle through redistribution of the RNA-binding protein fragile X mental retardation 1 (FMR1) towards the ESCRT-0 complex [85,86].

Gasdermin D (GSDMD) is a downstream effector of the NLRP3 inflammasome and a substrate for caspase-1. Upon cleavage by caspase-1, its N-terminal fragment form a large pore in the plasma membrane that mediates the release of IL-1β and IL-18 and in addition is responsible for pyroptotic cell death [87]. GSDMD has been s also found to be involved in the release of microparticles enriched with both of IL-1β and P2X7 following the activation of the P2X7 receptor [88]. In contrast, another study reported that, upon stimulation with LPS and eATP, the Ras GTPase-activating-like protein IQGAP1 acted as a molecular adaptor mediating the association of GSDMD to the ESCRT system and thereby promoting the biogenesis of IL-1β-containing exosomes in response to activation of the NLPR3 inflammasome [89]. In this context, stimulation with LPS and eATP activated the NLRP3 inflammasome without inducing pyroptotic cell death.

The metabotropic ATP/UTP-sensitive P2Y_2_ receptor has been shown to be involved in IL-1β release through caspase-1-dependent inflammasome activation in breast cancer. It was reported that activation of the P2Y_2_ receptor was associated with tumour growth and with the release of higher levels of MMP-9 and IL-1β *in vivo* [90]. In this study, it was demonstrated that the human MDA-MB-231 breast cancer cell line exhibited inflammasome activity following treatment with TNF-α and ATP. This was evidenced by measuring caspase-1 activity and IL-1β secretion. This effect was significantly reduced by knocking-down the expression of P2Y_2_ [90]. These results are really intriguing since P2Y_2_ down-stream signalling pathway is not known to activate the cascade responsible for inflammasome activation. In same cells, the use of extracellular ATP was demonstrated to increase intracellular Ca^2+^ concentration and this way activate large conductance Ca^2+^-sensitive K^+^ (BK_Ca_) channels [91]. Therefore, one may speculate that P2Y_2_ could indirectly activate the inflammasome by activating Ca^2+^-sensitive K^+^ channels responsible for a K^+^ efflux. This should be further investigated.

In conclusion, the inflammasome emerges as an important modulator for the secretion of both IL-1β and IL-18, as well as for that of extracellular vesicles, thereby mediating multiple immunoregulatory and cancerous responses. Nevertheless, the mechanistic ways by which inflammasomes control secretion of EVs and whether all possible inflammasome activators enhance secretion of EVs, are still unsolved questions.

## 5 P2X7 receptor and EV release from immune and cancer cells

In immune cells, eATP is one of the classical stimuli that trigger the release of EVs to the extracellular space from dentridic cells (DCs) [92], microglia [93] and macrophages [94] through activation of the P2X7 receptor. Indeed, Mackenzie and colleagues were the first to study this phenomenon and dissociate the role of the P2X7 receptor in the formation of membrane blebs and microvesicle shedding from the induction of apoptosis in monocytes [25]. Upon P2X7 receptor activation occurred a rapid phosphatidyl serine (PS) flipping to the outer leaflet of the plasma membrane and the formation of blebs. These events were accompanied with the release of PS-positive-microvesicles. Both bleb formation and PS exposure are usually considered as markers of apoptosis. However, in this case it was demonstrated that the release of microvesicles was induced within a few minutes after receptor activation and that blebs formation and PS flip were completely reversible after the stimulation was arrested and did not trigger cell death [26]. It was proposed in the aforementioned study that P2X7-induced microvesicles shedding acts as a secretory pathway for the rapid release of IL-1β into the extracellular compartment [25]. While the mechanism of microvesicle shedding was not clearly elucidated so far, it has been suggested that an interaction between Src homology 3 (SH3) domain of Src-kinase and the SH3 binding motif present in the C-terminal domain of the P2X7 receptor could lead to the phosphorylation of p38 mitogen-activated protein kinase (p38 MAPK) upon receptor stimulation [95,96]. It is also known that phosphorylated p38 can activate and stimulate the translocation of A-SMase from the lysosomal lumen to the outer leaflet of plasma membrane where it hydrolyses sphingomyelin to ceramide, thus facilitating the formation and release of large EVs [56]. Moreover, the P2X7 receptor can trigger F-actin cytoskeletal reorganization through Rho-associated protein kinase (ROCK), a pathway indispensable for membrane blebbing in macrophage [97] (Fig. 2).

Treatment of THP-1-derived macrophages with atmospheric particular matters in a size of 2.5 nm (PM_2.5_) significantly increased the release of exosomes via activation of the P2X7 receptor, which was markedly decreased by treatment with P2X7 antagonists. Consequently, exosomes were found to transfer PM_2.5_ to recipient macrophage cells stimulating the secretion of tumour necrosis factor (TNF)-α and IL-1β [98]. It has been demonstrated that stimulation of the P2X7 receptor induced the release of active TNF-α converting enzyme (TACE) in macrophage-derived exosomes by increasing intracellular Ca^2+^ level and phosphorylation of p38 MAPK that facilitates shedding of plasma membrane-bound TNF-α from macrophages [99].

The tumour microenvironment (TME) is well-known to promote local metabolic stress such as hypoxia/ischemia and release of molecules that regulate cancer progression and metastases. Extracellular ATP is one of the main components present in the TME, that can be hydrolysed to adenosine by ectonucleotidases. These are known affect cancer cell proliferation, migration, matrix invasion and mediate immunosuppression [100]. Interestingly, several studies have reported that cancer cells release much more exosomes than normal cells, particularly under metabolic stresses like hypoxia [35,101]. Indeed, the interconnection between eATP, purinergic signalling and release of EVs in the TME has become a new research field of interest.

Among all purinoreceptors, the P2X7 receptor is the mostly studied purinergic receptor in cancer [7,[102], [103], [104], [105]]. The implication of this receptor in the release of EVs from neuroblastoma cancer cells was firstly demonstrated by Gutiérrez-Martín et al. [106]. They indicated that the P2X7 receptor regulated Ca^2+^-dependent exocytosis of vesicles containing ATP, thus possibly responsible for a positive feedback mechanism. The role of the P2X7 receptor in the Ca^2+^-dependent secretion of small EVs was confirmed in melanoma [107] and breast cancer [108] cells. Notably, inhibition of either the ectopic ATP synthase or the P2X7 receptor suppressed the release of exosomes from cancer cells [109]. In intrahepatic cholangiocarcinoma HuCC-T1 cell line, downregulation of the *P2RX7* expression, due to the hypermethylation of its promoter consequently to the transfection of cells with a mutant isocitrate dehydrogenase gene (IDH1^R132C^), attenuated the levels of tetraspanins CD9 and CD81 in supernatants. These results further suggest an important role of the P2X7 receptor in the secretion of exosomes by cancer cells [110].

## 6 P2X4 receptor and EV release

As previously discussed, MVBs can be fused to lysosomes for degradation or fused to the plasma membrane leading to the extracellular release of small EVs [35]. Therefore, the impairment of lysosomal function can lead to increased release of small EVs [111]. Conversely, autophagic activity decreases small EV release, since autophagosomes can be merged with MVBs to form amphisomes and subsequent degradation by lysosome [112]. However, the interplay between exosome biogenesis and autophagy appears to be more complex, as the autophagic pathways mediate release of exosomes through the fusion of amphisome with the plasma membrane, depending on the stress conditions [112].

The P2X4 receptor is mainly expressed in the endosomal compartment, particularly in late endosome, lysosome and lysosome related organelles (LRO) [113]. The P2X4 receptor plays a major role in the fusion of endolysosomal compartment mediated by the release of endolysosomal Ca^2+^ into the cytosol and subsequent activation of calmodulin [114] and the fusion of LRO with the plasma membrane [113]. In a previous work, we showed the contribution of the P2X4 receptor in mammary cancer invasion and progression via inducing release of lysosomal cathepsin D into the extracellular matrix and promoting autophagy-mediated epithelial–to-mesenchymal transition (EMT) [115]. Recently, it has been also reported that activation of P2X4 by piperine increased autophagic flux by enhancing the autophagosome-lysosome fusion [116]. Taken together, these studies support a possible role of the P2X4 receptor in either small EV secretion or degradation, through the control of endo-lysosomal fusion to different compartments depending on the microenvironments and metabolic contexts. Nevertheless, the exact mechanism by which the P2X4 receptor regulates small EV release or degradation, either through a canonical or an autophagic process, has not been clearly identified so far. It has been proposed that P2X4 and pannexin-1 are directly involved in the secretion of miRNA-containing exosomes by HCV infected hepatocytes [117]. In this study, it was shown that pannexin-1 cleavage by caspase-3 resulted in the stimulation of plasma membrane P2X4 and subsequent Ca^2+^ influx, leading to activation of *n*-SMase and release of small EVs. However, the ATP concentration used for the stimulation of the P2X4 receptor in this study could activate other purinergic receptors, especially the P2X7 receptor, questioning the specificity of the pathways involved.

Accumulating evidence also supports the contribution of the P2X4 receptor in activation of the NLRP3 inflammasome. Notably, in patients suffering from diabetic nephropathy, there is a positive linear correlation between the expression of the P2X4 receptor in renal tubule epithelial cells and the urinary secretion of both IL-1β and IL-18. Moreover, the P2X4 receptor was co-localized with NLRP3, IL-1β, and IL-18 in renal tubule epithelial cells of patients with type 2 diabetic nephropathy. These results were confirmed *in vitro* using HK-2 proximal tubule kidney epithelial cells treated with high concentrations of glucose. These observations suggest the involvement of an eATP-P2X4 signalling pathway in activation of the NLRP3 inflammasome by high concentrations of glucose, consequent release of cytokines from the IL-1 family and development of tubulointerstitial inflammation [118]. Hung and collaborators reported that treatment of gingival epithelial cells with eATP stimulated the generation of ROS via a multiprotein complex composed of P2X4, P2X7 receptors and pannexin-1, which can activate the NLRP3 inflammasome and caspase-1. Inhibition or depletion of one of these proteins from the complex inhibited the release of IL-1β, suggesting a positive regulatory role of P2X4 in inflammasome activation [119]. Furthermore, it was demonstrated that the anti-parasitic drug ivermectin, that allosterically activates the P2X4 receptor, induced cell death via both apoptosis and necrosis in mouse (4T1) and human (MDA-MB-231) triple-negative mammary cancer cell lines. This cell death was mainly due to the activation the P2X4/P2X7/pannexin-1 complex by eATP and associated with activation of caspase-1, thus consistent with pyroptosis [120]. Furthermore, in a mouse model of allergic airway inflammation, it was demonstrated that either deficiency of the P2X4 receptor or treatment with the specific P2X4 antagonist 5-(3-Bromophenyl)-1,3-dihydro-2H-benzofuro[3,2-e]-1,4-diazepin-2-one (5-BDBD), alleviated airway inflammation. Notably, there was a significant decrease in the production of IL-1β in *P2rx4*-deficient bone marrow-derived dendritic cells that was accompanied by a marked decreased the expression level of the P2X7 receptor [121]. Recently, in an animal model of Parkinson's disease, it was shown that either knock-down or antagonism of the P2X4 receptor significantly decreased the secretion of both IL-1β and IL-18 in dopaminergic neurons. These results further suggest the implication of the P2X4 receptor and associated signalling pathway in activation of the NLRP3 inflammasome [122]. Furthermore, these accumulating results strongly highlight the interactions between P2X4 and P2X7 receptors for activation of the NLRP3 inflammasome. Therefore, it is tempting to hypothesize that the P2X4 receptor, like the P2X7 receptor, is involved in inflammasome-dependent release of EVs.

## 7 Ectonucleotidases CD39/CD73, adenosine receptors and EV release

The role of CD39 and CD73 nucleotidases in the generation and release of EVs is poorly understood. However, tumour-derived exosomes (TDE) and microvesicles are well-known to contain CD39 and CD73, which can promote local production of adenosine and bear important immunosuppressive activities. Clayton et al. reported the generation of adenosine by exosome CD39 and CD73 from several human cancer cell lines and the resulting inhibition of T cells through activation of the A2A adenosine receptor [123]. Interestingly, exosomes isolated from plasma of head or neck cancer (HNSCC) patients presented higher CD73 activity and induced significantly more adenosine production than did exosomes from control patients [124]. Furthermore, a positive correlation has been identified between the CD39/CD73 levels in plasma-derived exosomes and the higher stage of HNSCC patients [125]. CD73-expressing exosomes derived from U-87 MG glioblastoma cell line inhibited aerobic glycolysis of T-cells through the A2A adenosine receptor, thus promoting tumour growth *in vivo* [126]. Furthermore, adenosine generated by CD73-expressing exosomes from pancreatic and lung cancer cells has been reported to activate the A3 receptors in mast cells, thus upregulating the expression of angiogenic genes encoding for IL-6, IL-8 and VEGF, which in turn promoted tissue remodelling and tumour growth [127]. CD39/CD73-expressing TDE also promoted angiogenesis through activation of the A2B receptor in UMSCC47 head or neck cancer cells [128]. Similarly, microvesicles from bone marrow of multiple myeloma patients were shown to carry more CD39/CD73 and produce more adenosine than those from control patients, also supporting the contribution of large EVs to the immunosuppressive effects [129].

The direct implication of adenosine receptors in the regulation of exosome production has recently been demonstrated by Ludwig and collaborators in different cell lines. In preglomerular isolated rat vascular smooth muscle cells (PGVSMCs), activation of the A1 and A2A receptors was found to decrease exosome release under both basal and metabolic stress conditions, whereas activation of the A2B receptor impaired exosome release only under metabolic stress conditions. Exosome production from U251 glioblastoma, Mel526 metastatic melanoma and MDA-MB-231 breast cancer cell lines were significantly reduced by the selective A2A receptor agonist CGS 21680. However, cells treated with cisplatin and the selective A2A receptor antagonist SCH 442416 stimulated exosome production under metabolic stress [130]. Activation of the A2A receptor also induced apoptosis in Jurkat cells and subsequent release of immunosuppressive exosomes enriched with Fas-associated death domain (FADD) and, conversely, treatment with the A2B receptor antagonist MRS1754 reduced exosome production. These results have led to the conclusion that A2A suppresses exosome release in all investigated cell lines, whereas the effects of A1 and A2B on exosome release might be more specific to cell types and environmental conditions. Furthermore, activation of the A2B receptor decreased, whereas activation of the A3 receptor increased, the release of FADD-containing microvesicles, respectively. Both receptors were found to regulate apoptosis through FADD ligand [130,131].

## 8 Conclusions

The purinergic signalling represents a very powerful signalling pathway involving different receptors that are often expressed in the same cells, rendering it very complex to study. Furthermore, these receptors (seven P2X isoforms forming multiple heteromeric and heterotrimeric receptors, eight P2Y receptors and four adenosine receptors) which exhibit different ligand selectivity and sensibility, can be activated simultaneously in a single cell type, thus activating different secondary messengers and pathways. The half-life of ligands is relatively short and their levels in tissues are difficult to monitor. Furthermore, the activity of ectonucleotidases leads to generation of other nucleotides or nucleosides, therefore switching to the activation of other receptors that might generate opposite or modulatory responses. These features add to the complexity of analysing the purinergic signalling in a time-scale manner, and make it even more complicated to understanding of its consequences at the level of a multicellular tissue or organ. However, until recently it was considered that the purinergic signalling was a short-reach paracrine cell-to-cell communication, because of the hydrolysis of nucleotides and nucleosides by ectonucleotidases.

It is now very clear that actors of the purinergic signalling, receptors and ectonucleotidases, are important contributors of distance communications through the control of EV biogenesis, degradation, or even its content. The purinergic signalling mechanisms discussed in this review have mainly come from cancer or immune cells. However, it can be postulated that some if not all of these mechanisms function in other cell types and are activated in some specific environmental contexts such as in ischemic, metabolic, mechanical or infectious stress conditions.

From the published results, it appears that ionotropic ATP-gated P2X4 and P2X7 receptors and metabotropic ADP-sensitive P2Y_1_ and P2Y_12_ receptors might be involved in the release of either large or small EVs in response to luminal or extracellular ATP, while CD39 and CD73 might be more prominently expressed on the surface of EVs thus controlling the relative levels of ATP/adenosine in the surrounding environments. Nevertheless, more studies are needed to fully understand the mechanistic pathways involved following P2 receptor activation and whether they could specifically modulate the release of a specific subtype of EVs. Also, it would be of interest to monitor the presence of such receptors in EVs and whether they could also be transferred, similarly to the EV content, as they might generate new signals and induce new phenotypes in recipient cells.

## Funding

This work was supported by the "Ministère de la Recherche et des Technologies", the Région Centre-Val de Loire (grant “CanalEx” to S.R., grant “ExoGral” to S.R.), the Institut National du Cancer (grant INCA_16110 “PURINO4EXO” to S.R.), the FHU SUPORT (grant “DREAMT” to P.G., grant “CodEx Rein” to S.R.), the ADRIC Association in Tours (France).

T. D. was recipient of a PhD grant from the Région Centre-Val de Loire. M. E. was recipient of a Post-doctoral grant from the Institut National du Cancer. L-H.J. was recipient of an Invited Professorship from the University of Tours.
